# Comparison of diagnostic accuracy between periapical and panoramic radiographs and cone beam computed tomography in measuring the periapical area of teeth scheduled for periapical surgery. A cross-sectional study

**DOI:** 10.4317/jced.55986

**Published:** 2019-08-01

**Authors:** Amparo Ramis-Alario, Beatriz Tarazona-Alvarez, Juan Cervera-Ballester, David Soto-Peñaloza, Miguel Peñarrocha-Diago, David Peñarrocha-Oltra, María Peñarrocha-Diago

**Affiliations:** 1 Professor of Master in Oral Surgery and Implantology, Faculty of Medicine and Dentistry, University of Valencia, Valencia, Spain; 2 Assistant Professor of Orthodontics, Master in Orthodontics, Faculty of Medicine and Dentistry, University of Valencia, Valencia, Spain; 3 Chairman of Oral Surgery. Director of the Master in Oral Surgery and Implantology, Faculty of Medicine and Dentistry, University of Valencia, Valencia, Spain; 4 Assistant Postdoctoral Professor of Oral Surgery, Master in Oral Surgery and Implantology, Faculty of Medicine and Dentistry, University of Valencia, Valencia, Spain; 5 Full Professor of Oral Surgery, Master in Oral Surgery and Implantology, Faculty of Medicine and Dentistry, University of Valencia, Valencia, Spain

## Abstract

**Background:**

The aim of the study was compare the sensitivity and measurements obtained from teeth with apical lesions scheduled for periapical surgery using three different diagnostic methods: periapical radiography (Gendex Expert DC), panoramic radiography (Planmeca® Promax 3D Classic) and cone beam computed tomography (CBCT) (Planmeca® Promax 3D Classic).

**Material and Methods:**

This cross-sectional study involved 35 patients (45 teeth) scheduled for periapical surgery in which periapical radiographs, panoramic radiographs and CBCT scans had been obtained. The images were used to analyze the maximum vertical and horizontal dimension and the resulting areas of the periapical lesions based on the three diagnostic methods.

**Results:**

The two-dimensional techniques (periapical radiography and panoramic radiography) yielded a sensitivity of 82% versus 100% in the case of CBCT. The mean vertical dimension of the apical areas was 5.48 mm with periapical radiography and 5.04 mm with panoramic radiography – the difference with respect to CBCT being statistically significant (6.36 mm for the coronal sections). There were no significant differences among the three techniques in terms of horizontal dimension (p>0.05) or lesion area.

**Conclusions:**

The sensitivity of periapical radiolucencies detected using CBCT was significantly greater than with the two-dimensional imaging techniques. Significant differences between the latter and CBCT were only observed in the case of the vertical measurements.

** Key words:**Periapical lesion, apicoectomy, CBCT, periapical radiography, panoramic radiography.

## Introduction

Apical periodontitis is defined as radiolucency associated to the most apical portion of the root of the tooth and measuring at least twice as wide as the normal periodontal width (1). Such lesions are usually accompanied by bone reabsorption, producing a radiographically identifiable periapical area (2).

Apical periodontitis is monitored based on the clinical and radiographic findings. Radiological assessment is essential for the successful and timely diagnosis, since the condition may be asymptomatic (3,4). The prevalence of apical periodontitis is greater in teeth that have been subjected to endodontic treatment (5), being observed in 36% of all endodontically treated teeth, depending on the population studied (6).

In relation to the radiological study of periapical lesions, a number of authors have shown cone beam computed tomography (CBCT) to offer greater diagnostic sensitivity than periapical radiography (4,7). The fact of being able to see the images in three dimensions improves and advances the diagnosis (8). However, the few studies that have compared the dimensions of the periapical radiolucency using two-dimensional radiography and CBCT have reported no significant differences between the two techniques (9,10).

On the other hand, despite the widespread use of panoramic radiography in dental clinics, no studies have established quantitative comparisons of the areas obtained with two-dimensional radiography and CBCT. Furthermore, both bidimensional radiographs, have important limitations, as they are overlays of certain anatomical structures, the fact that lesions limited to cancellous bone can go unnoticed, until the cortical bone is eroded or neither these lesions will be detected until the bone mineral loss reaches 30-50% (1).

Studying the previous size of the periapical lesion is important because it is strongly related to the prognosis of the apical surgery. Higher rates of successful are guaranteed with lesions minors than 5 mm, versus higher lesions, hence the importance of studying the size before apical surgery (11,12).

In keeping with the above-mentioned observations, the present study compares: (i) the sensitivity to detect apical lesions in teeth scheduled for periapical surgery and (ii) the size of these lesions, using three different radiographic diagnostic techniques: periapical radiography, panoramic radiography and CBCT. The null hypothesis is that there is no difference neither in the sensibility nor in none of the measurements between the three methods.

## Material and Methods

-Study design and participants

This study was conformed to STROBE guidelines for cross-sectional human observational study (13). The study was carried out in our Dental School (Valencia University, Valencia, Spain) between March 2015 and March 2017. All patients scheduled for periapical surgery were included. Written informed consent was obtained from all participants, and the study was approved by the local Ethics Committee (Reference: H1490891777899).

-Selection of cases

Patients with teeth scheduled for periapical surgery, with the obtainment of periapical radiographs, panoramic radiographs and CBCT scans. The exclusion criteria were: images of deficient quality (incomplete visualization of the roots or cortical components) and images with the three techniques obtained in a period of time superior to two months.

-Radiographic examination

The digital periapical radiographs were obtained with the Gendex Expert DC system (Gendex Dental Systems, Hatfield, USA), using the Vista Scan radiological sensor (Dürr Dental AG, Bietigheim-Bissingen, Germany) (4 x 3 cm) with the following exposure parameters: 65 kV, 7 mA, and times from 0.08 to 0.18, depending on the tooth evaluated. All the periapical radiographs were obtained using a parallel technique (Super-Bite, Kerr). The digital images were processed and filed using DBSWIN software (Dürr Dental). The panoramic radiographs were obtained with a Planmeca® system (Promax 3D Classic, Helsinki, Finland), using the Planmeca Romexis program for image processing. The exposure parameters were: 68 kV, 10 mA, 19 seconds.

The CBCT scans were obtained with a Planmeca® system (Promax 3D Classic, Helsinki, Finland), using the following operating parameters: 90 kV, 10 mA, 15 seconds for 180º rotation, and a voxel size of 0.15 mm. The field of view (FOV) of the detector panel was 40 x 40 mm. Planmeca Romexis Viewer software was used to process the CBCT images. Slice thickness was 0.20 mm. For each tooth the sections were modified to align the root axis with the vertical plane in the coronal and sagittal views.

The CBCT scans had been requested to precisely evaluate the anatomical structures affected and the endodontic conditions of the teeth, with a view to performing periapical surgery, and were not obtained as a routine study – thereby complying with the ALARA (As Low As Reasonably Achievable) criterion. The images were evaluated by two blinded examiners (both certified oral surgeons, J.C. and A.R.) not related to the treatment and follow-up of the patients.

-Calibration of examiners

Cone beam computed tomography images were used for calibration and evaluation of inter-examiner accuracy, measuring the distance from the apex of the mesial root of the first mandibular molar to the upper margin of the mandibular canal (14). Ten random measurements were obtained by both examiners, yielding a difference between them of 0.14 mm per image (range 0.0-0.28 mm). The level of agreement between reviewers is obtained through de intraclass correlation coeficient (ICC) and interpreted according the Landhis and Koch scale (15) .

-Evaluation of images

In the global study, each measurement was made by the two examiners, with calculation of the average of both measurements. This was the value subsequently used in the data analysis, except in those cases where the two measurements differed by more than 0.2 mm. In such cases a third measurement was made jointly by both examiners (14). The measurements were made in three sessions spaced one week apart. In each session one-third of the images obtained with the different radiographic techniques were evaluated on a random basis (7).

-Linear and area measurements

The two-dimensional (2D) radiographs and CBCT scans were displayed on a Full HD monitor with a resolution of 1920 x 1080 pixels in a dimly lit room. The periapical lesions were measured as follows: Vertical measurements: the maximum vertical dimension (in mm) of the area was recorded. Horizontal measurements: the maximum dimension (in mm) perpendicular to the previously obtained vertical measurement was recorded.

**Figure 1:**
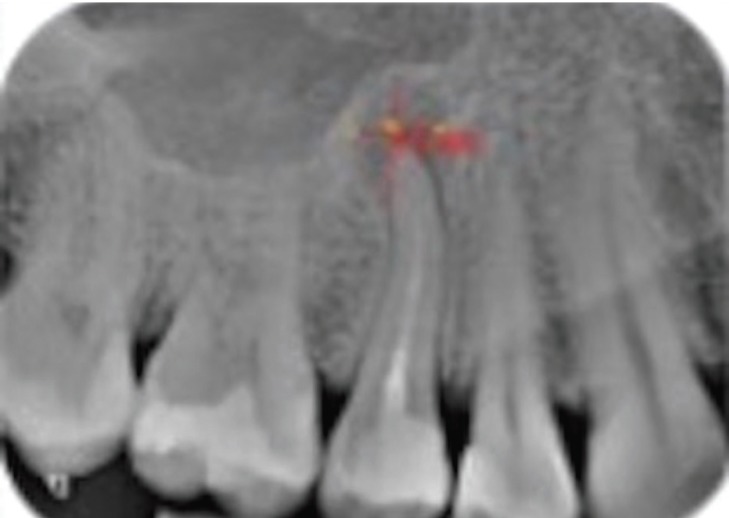
Vertical and horizontal measurements obtained by periapical radiography.

**Figure 2:**
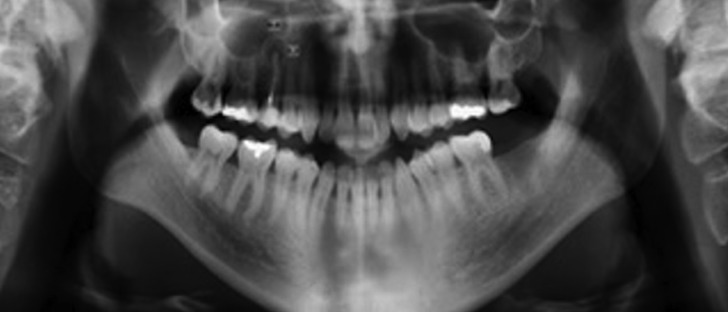
Panoramic radiograph with the measurements made on the radiolucent area of tooth 15.

**Figure 3:**
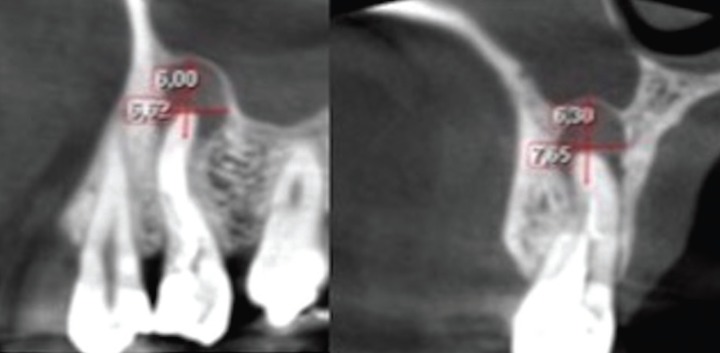
Sagittal (A) and coronal (B) sections used to obtain the measurements of the periapical area of tooth 15.

The same measurement protocol was used for periapical radiography (Fig. 1), panoramic radiography (Fig. 2) and CBCT (Fig. 3^3^). In the latter technique the measurements were obtained from the sagittal and coronal sections. The corresponding area was calculated as a rectangle (vertical measurement multiplied by horizontal measurement), according to a previous study (9). The examiners were able to modify size, brightness and contrast of the images of all three techniques.

-Statistical analysis

Inter-examiner agreement was assessed by contrasting the normal distribution of the differences in measurements using the Kolmogorov-Smirnov test, with calculation of the coefficient of variation (CV) and the intraclass correlation coefficient (ICC). The Student t-test for dependent samples was used to compare the diagnostic accuracy of the three radiological techniques. The Mann-Whitney U-test was used to assess the impact of patient gender upon lesion size, while the Spearman nonlinear correlation coefficient was used to evaluate the influence of age. The level of statistical significance was defined as 5% (α=0.05). The statistical analysis was conducted using statistical program R (Version R 3.5.1).

## Results

-Sample characteristics and descriptive data

The initial sample size consisted of 82 patients, of which 47 were excluded after application of the inclusion and exclusion criteria. Patients excluded from the study, with reasons were:

1. Patients with periapical or panoramic radiographs, or CBCT scans, not allowing full visualization of the roots and cortical component of the affected teeth (n=29).

2. Poor image quality (n=4).

3. Time intervals among the three radiographic studies > 2 months (n=6).

4. Images taken after the apicectomy (n=8).

The final study sample therefore consisted of 35 patients (45 teeth): 18 males and 17 females, with a mean age of 47 years (range 19-74). The upper incisors were the teeth most frequently affected by periapical disease. The distribution of the teeth is shown in  Table 1.

**Table 1:**
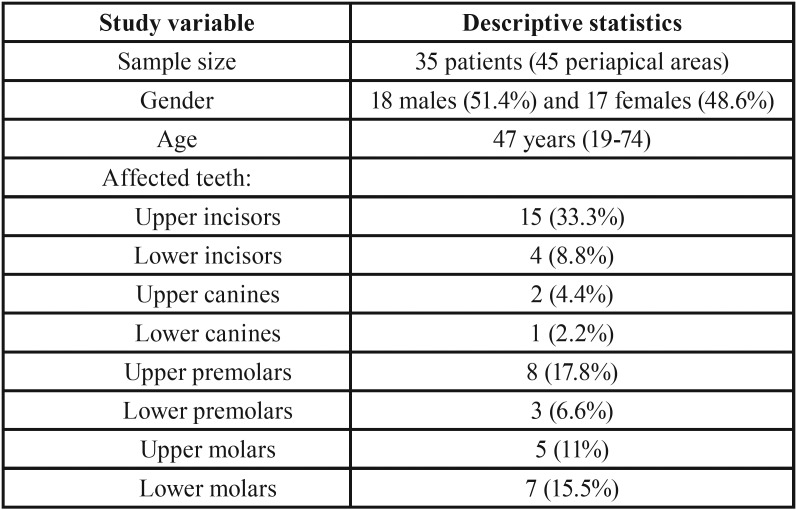
Description of the study sample.

-Detection of periapical areas

The conventional two-dimensional techniques (periapical radiography and panoramic radiography) both showed a sensitivity of 82%. Of the 45 periapical areas, 8 were not detected by either technique: two corresponded to the anterior sector (incisors and canines) and 6 to the posterior sector (premolars and molars). In percentage terms, the two-dimensional techniques failed to register 4.5% of the anterior lesions and 13.5% of the posterior lesions. In contrast, CBCT registered all 45 radiolucent areas. Inter-examiner agreement was almost perfect, with CV < 3% and ICC > 0.99 in all cases.

-Vertical measurements of periapical areas

The mean vertical measurement of the apical area was found to be 5.48 mm with periapical radiography (n=44, SD±3.93) and 5.04 mm with panoramic radiography (n=45, SD±3.61 mm). The results in the case of CBCT were 6.36 mm in the coronal sections (n=45, SD±3.65) and 6.38 mm in the sagittal sections (n=45, SD±3.45). There was a lower molar which wasn´t measured on periapical radiographs because the distal root was not available on the image, so the sample size is different in this method with regard to the other two. There were no significant differences between the two-dimensional techniques (periapical radiography and panoramic radiography), though significant differences were observed in the vertical measurements between the two-dimensional techniques and CBCT, with higher values when the latter technique was used (*p*<0.05) ( Table 2).

**Table 2:**
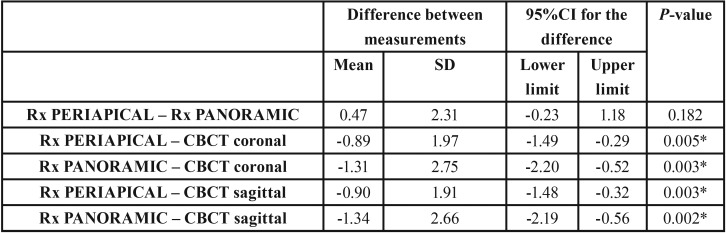
Difference in measurements (mean and standard deviation [SD]), 95% confidence interval (95%CI), t-test for dependent samples (*p*-value) referred to the vertical dimension (height) among the different radiographic techniques.

-Horizontal measurements

With regard to the horizontal measurements, the mean values were 4.80 mm in the case of the periapical radiographs (n=44, SD±3.43) and 5.01 in the case of the panoramic radiographs (n=45, SD±3.91). In the case of CBCT the values were 5.63 mm in the coronal sections (n=45, SD±2.95) and 5.10 mm in the sagittal sections (n=44, SD±2.26). No significant differences were observed on comparing the different radiographic techniques: all three were therefore considered to offer similar results in relation to the horizontal measurements (*p*>0.05) ( Table 3).

**Table 3:**
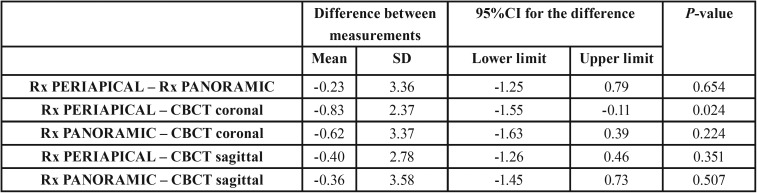
Difference in measurements (mean and standard deviation [SD]), 95% confidence interval (95%CI), t-test for dependent samples (*p*-value) referred to the horizontal dimension (width) among the different radiographic techniques.

-Area of periapical bone defects

With regard to the calculation of lesion area from the vertical and horizontal measurements, the results were 37.33 mm2 for the periapical radiographs (n=44, SD±44.49) and 36.37 mm2 for the panoramic radiographs (n=45, SD±47.63). In the case of CBCT the area was 44.76 mm2 in the coronal sections (n=45, SD±49.32) and 36.59 mm2 in the sagittal sections (n=44, SD±29.41). Likewise in this case there were no significant differences among the different radiographic techniques: all three were therefore considered to offer similar results in relation to the area measurements ( Table 4). The sample size differs from one unit between the different methods, because there was an area, due to its size, in periapical radiography and in sagittal sections it couldn´t be measured, as its margin was not observed completely so vertical and/or horizontal measure couldn´t be registered. Lastly, no significant associations were found between lesion area and patient age or gender.

**Table 4:**
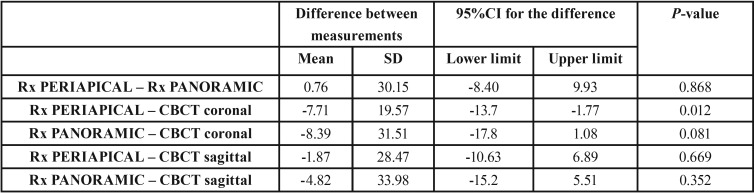
Difference in measurements (mean and standard deviation [SD]), 95% confidence interval (95%CI), t-test for dependent samples (*p*-value) referred to lesion area among the different radiographic techniques.

## Discussion

The present study compared the three radiodiagnostic techniques currently used for the measurement and analysis of radiolucencies produced by periapical infection. Specifically, we compared the different vertical and horizontal measurements and areas obtained with the three imaging techniques, and assessed their diagnostic validity and the possible influence of other associated factors such as patient gender and age.

-Sensitivity of three methods

Is well known that CBCT sensitivity for the prevalence of periapical radiolucencies was 100% (4,16). The findings of the present study are in agreement with the above-mentioned trend. However, the prevalence decreased to 82% when using the two-dimensional radiographic techniques (periapical radiography and panoramic radiography). The failure rate in identifying periapical lesions with these techniques in posterior and anterior teeth was 13.5% and 4.5%, respectively. Liang et al. (17) recorded a similar percentage of 5% in anterior teeth with periapical radiography, thus coinciding 

with our own observation that anterior sector lesions are more easily visualized than posterior lesions. No significant associations were found between lesion area and patient age or gender.

The literature offers similar data (14,16), with a higher periapical lesion non-visualization rate associated to two-dimensional imaging techniques. Lesions close to the maxillary sinus are very likely to go undetected with two-dimensional methods, due to overlapping – this situation being even more probable in the case of the upper second molars (18). Furthermore, CBCT is able to identify possible oroantral communications, which may be important for the planning and success of surgery.

Only one study to date has assessed sensitivity in the case of panoramic radiography (19), though the reported figure (28%) falls far short of our own 82%. The difference may be explained by a number of factors, such as the fact that the mentioned study considered teeth both with and without endodontic treatment; as a result, in the case of a tooth not subjected to endodontic treatment, the examiner might not notice the periapical area in the absence of important coronal destruction.

 The results of the present study confirmed that both the vertical and horizontal measurements were comparatively greater with CBCT than with the two-dimensional techniques, though statistical significance was only reached in the case of the vertical measurements. These findings are consistent with the data found in the literature, which likewise describe CBCT as being more accurate than periapical radiography (1,2,7,17,19–24) or panoramic radiography (18,19,25). All these studies are limited to the evaluation of the sensitivity of the techniques, and only a few have compared the capacity of the three imaging methods to demonstrate the full extent of the lesions, measuring and comparing the lesion areas (9,10,26,27).

-Radiographic and CBCT measurements

We recorded no significant differences in the vertical measurements of the lesions between the two-dimensional radiographic techniques, though significant differences were observed on comparing these techniques with CBCT. However, Bornstein *et al.* (2) recorded no significant differences on comparing the maximum diameter of the areas with the different radiological methods (e.g. CBCT and periapical radiography).

Similar findings were reported by Gouveilla *et al.* (10). This coincides with the observations of all those studies that have examined these associations (1,28–30).

-Limitations

However, some limitations need to be considered, such as the variability of vertical and horizontal magnification, factors that depends on patient position in panoramic radiographs, as well the position of periapical radiographs, was not taken into account as independent factors on analysis.

The clinical relevance of this study lies in the perspective that is not always affordable for a clinician to acquire a CBCT scan for its private´s practice, being this economic aspect or third-party services that suppose an important barrier for its accessibility, in particular in developing countries. Strikingly, this limitation is not proportional with the high demand of root canal treatments and prevalence of periapical lesions in everyday clinical practice. Thus, despite limitations, the results of the present study could be extrapolated to clinical practice for apical lesions diagnostic.

Two dimensional radiographic techniques showed significantly lower sensitivity to detect periapical radiolucencies lesions than CBCT. In relation to the vertical dimension of the apical lesions, significant differences were observed between the conventional two-dimensional techniques and CBCT – the former tending to underestimate vertical height. No differences in horizontal dimension or area were observed, however.
